# Editorial: Interplay between schools and society in the context of infectious disease spread

**DOI:** 10.3389/fpubh.2022.1018204

**Published:** 2022-09-14

**Authors:** Hasan Güçlü, Emine Yaylali

**Affiliations:** ^1^Department of Biostatistics and Medical Informatics, School of Medicine, Istanbul Medeniyet University, Istanbul, Turkey; ^2^Department of Industrial Engineering, Istanbul Technical University, Istanbul, Turkey

**Keywords:** COVID-19 pandemic, infectious diseases, statistical analysis, epidemics in schools, pandemic psychology

## Introduction

Educational systems, especially primary and secondary schools, have been considered hotbeds for infectious diseases, and their closures have been used as a major non-pharmaceutical intervention for the mitigation of infection risks (1). Since the WHO declared COVID-19 as a global pandemic, most countries closed down and later reopened schools and universities to reduce mixing and human mobility, which are contributing factors to infectious disease spread. UNICEF reports that about half of the world's population was affected by country-wide or local school closures in more than 60 countries (2). School closures not only epidemiologically but also psychologically affected their students and populations (3).

Studying infectious disease epidemiology in the school context could contribute to the overall study of infectious disease spread as well as its effect on the overall wellbeing of the students. The goal of this Research Topic is to collect and compile relevant research on the role of schools and universities in the spread and mitigation of infectious diseases, including the latest COVID-19 pandemic, and the role of infectious diseases and associated mitigation measures on school systems and students. First and foremost, for example, school closures have been found to be somewhat effective during influenza pandemics (4, 5) but the effect of closures on schools and students has not been investigated in great detail until the COVID-19 pandemic that started late 2019. There are in total eight articles on this Research Topic: two brief research reports, one community case study, three original research articles, and two perspective articles.

In one study, Perramon et al. investigated COVID-19 prevalence among school-age children in Catalonia, Spain, and compared it with the adults' prevalence to check if it can be used as an indicator for epidemiological surveillance at the population level. They found that children had a slightly lower incidence that increased by age than the general population and the test positivity was also lower than in adults. The researchers explain these by the mass screening and comprehensive contact tracing they administered and argue that school-age children could act as a proxy for the population.

In another study, Hatami et al. used an online parent-administered questionnaire on 704 high-school students in Iran to analyze the level of knowledge and safety practices as well as attitudes about COVID-19 during the first wave of the pandemic in March 2020. The respondents were found to have been informed about the pandemic at an acceptable level, although some lacked knowledge and showed unsafe practices that require addressing misinformation, especially in social and mass media.

Travis et al. studied the efforts of a college in Michigan, USA to stay open during the pandemic including wastewater surveillance, comprehensive testing, contact tracing, and isolation procedures from July to November 2020 in their perspective article. The researchers discuss lessons learned from these measures that require broad collaboration across multiple stakeholders in the college and emphasize the importance of a comprehensive, diverse, and cross-functional team overseeing these activities.

A perspective article by Brown discusses the future of schooling and schools in a post-pandemic world and how we could take advantage of the experiences gained around the globe. He specifically reflects upon an all-online education in which there exists two risks: lack of equitable access and dehumanization. Brown also argues that one can benefit from specific experiences that exist in different parts of the world.

One of the major problems adolescents face during the pandemic is school closure and mental wellbeing. Wiguna et al. investigated the mental health of 113 adolescents schooled at home during the early period of the pandemic in Indonesia in a cross-sectional study by a questionnaire. The researchers observed mental and behavioral problems at varying degrees compared to the pre-pandemic period.

Using face masks is the simplest and one of the most effective ways to protect others against a respiratory disease such as COVID-19. Hudaib et al. looked into the knowledge and awareness of masks and N95 respirators among 179 engineering students in Jordan in April 2020. The researchers found a moderate level of awareness of face masks and N95 respirators among students. The awareness of mask usage was higher among upper-level students than among younger ones but the awareness of the appropriate type of mask was lacking.

In a cross-sectional study by Qin et al. the researchers investigated the associations of COVID-19 risk perception, eHealth literacy, and protective behaviors among 5,641 university students in China through a survey administered between 10 and 15 July 2021. The results of this study show that there is still room for improvement in compliance with the public health measures, especially for younger males, and a low level of compliance could have contributed to sporadically occurring cases in the country despite strict regulations. This study also showed a high degree of positive correlation between eHealth literacy scores and positive protective behaviors.

Meng et al. investigated the spatial and temporal characteristics of influenza in schools over a period of 5 years (2014–2018) in Chongqing, China. In their study, they analyzed 111 preschool and school-based outbreaks, including 3,549 cases and their symptoms, and showed strong contrast between influenza type A and B. Their results have significant implications on the COVID-19 pandemic in which related variants co-circulate in society.

With more available data and technology as a scientific community, we have better tools and theoretical methodology to assess any kind of effect the schools might have on a pandemic and the effect of a pandemic on schools. These are multifaceted problems that require a multidisciplinary approach and this Research Topic serves as a starting point to discuss the wellbeing of our students and schools under the threat of pandemic infectious diseases.

## Author contributions

HG conceived the original concept of the Research Topic. HG and EY edited the papers and wrote this editorial together. Both authors contributed to the article and approved the submitted version.

## Funding

This work was partially supported by the Scientific and Technical Research Council of Türkiye through grant number 221S893.

## Conflict of interest

The authors declare that the research was conducted in the absence of any commercial or financial relationships that could be construed as a potential conflict of interest.

## Publisher's note

All claims expressed in this article are solely those of the authors and do not necessarily represent those of their affiliated organizations, or those of the publisher, the editors and the reviewers. Any product that may be evaluated in this article, or claim that may be made by its manufacturer, is not guaranteed or endorsed by the publisher.
